# Skin prick testing with extensively heated milk or egg products helps predict the outcome of an oral food challenge: a retrospective analysis

**DOI:** 10.1186/1710-1492-7-S2-A9

**Published:** 2011-11-14

**Authors:** Zein Faraj, Harold L Kim

**Affiliations:** 1 Michael G DeGroote School of Medicine, McMaster University, Hamilton, Ontario, Canada; 2 University of Western Ontario, London, Ontario, Canada

## Background

Children with milk and/or egg allergy can often tolerate heated forms of these foods. Skin prick testing (SPT) with commercial extracts followed by a possible oral food challenge (OFC) are routinely performed in these children. This study evaluated the clinical utility of a negative SPT with the real extensively heated milk or egg in predicting whether a child would tolerate an OFC to the heated food.

## Methods

Charts were reviewed in a single allergy clinic for any patient with a negative skin SPT to heated milk or egg, prepared in the form of a muffin. Data was collected on the success of the OFC to the muffin as well as age, sex, symptoms and co-morbidities in these patients.

## Results

Fifty-eight patients had negative SPT to the heated milk or egg in a muffin. All of these children underwent OFC to the appropriate heated food in the outpatient clinic. Fifty-five of these patients tolerated the OFC. The negative predictive value for the SPT with the extensively heated food product was 94.8%.

## Conclusions

SPT with heated milk or egg products was predictive of a successful OFC to the same food. Larger prospective studies are required to substantiate these findings.

